# Rational Development of Live-Attenuated Zika Virus Vaccines

**DOI:** 10.3390/pathogens12020194

**Published:** 2023-01-28

**Authors:** Awadalkareem Adam, Christy Lee, Tian Wang

**Affiliations:** 1 Department of Microbiology & Immunology, University of Texas Medical Branch, Galveston, TX 77555, USA; 2 John Sealy School of Medicine, University of Texas Medical Branch, Galveston, TX 77555, USA; 3 Sealy Institute for Vaccine Sciences, University of Texas Medical Branch, Galveston, TX 77555, USA; 4 Institute for Human Infections and Immunity, University of Texas Medical Branch, Galveston, TX 77555, USA; 5 Department of Pathology, University of Texas Medical Branch, Galveston, TX 77555, USA

**Keywords:** zika virus, live-attenuated vaccine, vaccine platform

## Abstract

Zika virus (ZIKV), a re-emerging mosquito-borne *flavivirus*, has caused outbreaks in Africa, Asia, the Pacific, and, more recently, in the Americas. ZIKV has been associated with the neurological autoimmune disorder Guillain–Barre syndrome in adults and congenital Zika syndrome in fetuses and infants, including microcephaly, spontaneous abortion, and intrauterine growth restriction. It is considered to be a major threat to global public health due to its unprecedented clinical impact on humans. Currently, there are no specific prophylactics or therapeutics available to prevent or treat ZIKV infection. The development of a safe and efficacious ZIKV vaccine remains a global health priority. Since the recent outbreak, multiple platforms have been used in the development of candidate ZIKV vaccines. The candidate vaccines have been shown to elicit strong T cell and neutralization antibody responses and protect against ZIKV infection in animal models. Some candidates have progressed successfully to clinical trials. Live-attenuated vaccines, which induce rapid and durable protective immunity, are one of the most important strategies for controlling flavivirus diseases. In this review, we discuss recent progress in the development of candidate live-attenuated ZIKV vaccines.

## 1. Introduction

Zika virus (ZIKV) is a re-emerging mosquito-borne positive sense RNA virus. It belongs to the *flavivirus* genus and the family of *Flaviviridae*, which includes several other known human pathogens such as West Nile (WNV), dengue (DENV), yellow fever (YFV), and Japanese encephalitis viruses (JEV) [1]. The virus was initially isolated from the blood of a rhesus monkey in the Zika forest in Uganda in 1947 [2]. It later caused outbreaks in the Island of Yap of the Federated States of Micronesia in 2007 [3] and in Pacific islands in 2013 [4]. In 2015, a large outbreak of ZIKV in Brazil was reported to be associated with Guillain–Barré syndrome and microcephaly in infants born to pregnant women [5,6]. The virus subsequently spread to the Americas and the Caribbean and caused more than one million human cases of infection [7]. ZIKV infection in humans results primarily from mosquito bites [8,9], sexual contact, and transmission via transplacental, blood transfusion, or organ transplantation [5,10,11]. Although the majority of human infection with ZIKV is asymptomatic and self-limiting, the virus is associated with severe neurological manifestations, such as Guillain–Barre syndrome in adults and congenital Zika syndrome (CZS) in fetuses and infants, including microcephaly, spontaneous abortion, and intrauterine growth restriction (IUGR) [12,13,14,15]. Currently, neither antiviral treatments nor vaccines are available for humans.

In recent years, multiple platforms have been pursued for ZIKV vaccine development, including the purified inactivated virus, DNA plasmid, mRNA, viral vector, and various types of live-attenuated vaccines (LAV). These candidate vaccines are reported to elicit high neutralizing antibodies and protect the host against subsequent ZIKV infection in preclinical studies, and some have progressed to clinical trials [16]. LAVs induce rapid and durable protective immunity. The LAV approach is one of the most effective strategies to control flavivirus diseases, as exemplified by the very successful YFV 17D and JEV SA14-14-2 vaccines. In this review, we discuss various approaches used to generate ZIKV LAV candidates in the recent preclinical studies with a focus on their effectiveness, immunogenicity, and safety and overview the progress made in the development of other platforms of ZIKV vaccines.

## 2. ZIKV Biology

**Figure 1:**
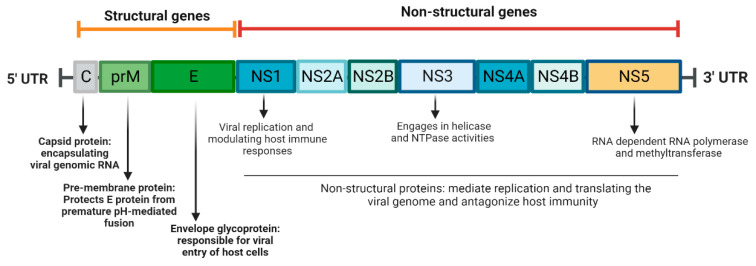
Schematic representation of Zika virus genome (created with BioRender.com on 22 January 2023).

The ZIKV genome is a single-stranded, positive-sense RNA molecule, approximately 11,000 nucleotides in length, with two untranslated regions and one open reading frame. The genome is translated and processed into ten proteins–three structural proteins [envelope (E), membrane (M), nucleocapsid), and seven nonstructural (NS) proteins (NS1, NS2A, NS2B, NS3, NS4A, NS4B, and NS5] [17] (Figure 1). Among the three structural proteins, the capsid protein is responsible for encapsulating the viral genomic RNA to form the nucleocapsid of ZIKV. The envelope (E) protein is primarily responsible for the host cell entry of the virus. Domain III within the E protein contains the epitopes for B and T cells, making it an important target for neutralizing antibodies to block virus infection [18,19]. The pre-M protein (prM), the precursor of the M protein, forms a heterodimer with the E protein to encapsulate the nucleocapsid within the endoplasmic reticulum. As the immature virion is transported to the Golgi and trans-Golgi Network, the prM protein protects the E protein from premature pH-mediated fusion. During maturation of the virus, proteolytic cleavage of the prM protein produces a mature M protein through furin protease cleavage [20,21]. The NS proteins are involved in replicating and translating the viral genome and antagonism of host immunity [17]. For example, NS1 is a 48 kDa protein that participates in the viral replication complex as well as modulates host immune responses. The NS3 protein engages in helicase and NTPase activities via its C-terminal domain, which is responsible for unwinding the genome RNA for transcription. The NS5 protein, another member of the viral replication complex, contains domains with RNA methyltransferase and RNA-dependent RNA polymerase activities [22].

## 3. Animal Models of ZIKV Infection

Animal models have been used to study ZIKV pathogenesis and to test vaccine efficacy. Mice are economical, readily available, and thus are commonly used. Systemic ZIKV infection in adult wild-type immunocompetent mice induces mild clinical symptoms with transient viremia and weight loss. Mice deficient in interferon (IFN)α/β receptor (Ifnar1^−/−^) alone or together with IFN-γ receptors with either C57BL/6 (AB6, or AGB6) or 129 genetic backgrounds (A129, or AG129) are much more susceptible to systemic ZIKV infection. Infected IFN-deficient mice exhibited weight loss and neurological symptoms and succumbed to infection within a two-week period [23,24,25]. In addition, several groups demonstrated that ZIKV infection at embryonic day 6.5 (E6.5) of the Ifnar1^−/−^ pregnant dams or wild-type pregnant dams treated with a blocking anti-Ifnar monoclonal antibody one day before infection recapitulate CZS in humans [26,27]. Non-human primates (NHP) are the natural host of ZIKV infection. Rhesus macaques (RMs), cynomolgus macaques (CMs), and pigtail macaques (PMs) are all susceptible to ZIKV infection [28,29,30,31,32]. The duration of viremia and tissue distribution of ZIKV RNA in infected macaques is similar to human infection. Compared to the murine model, the biology of macaque pregnancy and neural development are also closer to humans. ZIKV-induced fetal brain lesions and placental dysfunction and immunopathology were observed in pregnant PMs infected at either the second trimester or early third trimester [28,33] and in RMs infected at different stages of gestation, respectively [34].

## 4. Adaptive Immunity to ZIKV Infection and Vaccination

Animal models and clinical studies of ZIKV infection have provided important insights into host protective immunity. Humoral immune responses contribute to the control of ZIKV infection, viral dissemination, and ZIKV-induced diseases. Increased B cell activity has been reported in ZIKV-infected macaques [29,32,35,36]. A high titer of neutralization antibody developed within a week and continued for weeks following virus clearance in blood. Upon re-exposure to ZIKV, neutralization antibody titers were boosted [29,32,35]. T cells play a central role in adaptive immunity and are directly involved in viral clearance and/or provide help for B cells and antibody maturation during several other flaviviruses infections [37,38,39]. Lessons learned from clinical studies of several licensed flavivirus vaccines, including Dengvaxia and the YFV 17D vaccine, suggest that T-cell immunity is crucial for a safe, efficacious, and durable vaccine [40,41]. Upon ZIKV infection, CD8^+^ T cells contribute to virus clearance and host protection [42,43]. T cell epitopes are not only mapped in the structural protein but also in the NS proteins of flaviviruses. Many of the currently developed ZIKV vaccine platforms, including DNA plasmid, mRNA, and measles virus-vectored vaccines, are based on the constructs of the structural proteins, like PrM and E genes of ZIKV [44,45,46]. The candidate LAVs of ZIKV, which contain both structural and NS proteins, induce potent neutralization antibody and viral-specific CD4^+^ and CD8^+^ T cell responses in mice [47,48,49]. They are likely to lead to improved quality, quantity, and longevity of immunogenicity in ZIKV vaccine development.

## 5. ZIKV LAVs

**Figure 2:**
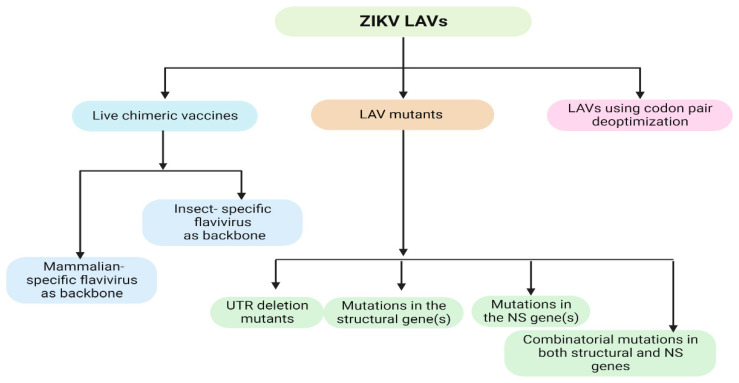
Schematic representation of current attenuation approaches to generate LAV ZIKV vaccines (created with BioRender.com on 22 January 2023).

LAVs mimic natural viral infections. They are known to induce long-lasting immune responses without the need for an adjuvant and a booster immunization. LAV represents one of the most effective strategies for flavivirus vaccine development, as exemplified by the success of YFV 17D and JEV SA14-14-2. A similar approach can be applied to ZIKV vaccine development. ZIKV infection causes congenital zika syndrome in pregnant women. Nevertheless, ZIKV vaccine trials have excluded pregnant women due to safety concerns [50]. One major challenge related to the safety of LAVs of flaviviruses is the inherent instability of the RNA genome [51,52]. Thus, the development of rational approaches for the attenuation of ZIKV is key to generating safe and efficacious ZIKV LAVs. Currently, there are three common approaches to generating LAV ZIKV vaccines: (1) making chimeric strains with an attenuated flavivirus backbone; (2) mutagenesis; (3) codon deoptimization (Figure 2).

### 5.1. Live Chimeric Vaccines

Chimeric flaviviruses are generated by replacing the structural protein genes of the backbone flavivirus with the corresponding genes of the target flavivirus. For a chimeric ZIKV vaccine, the backbone virus can be a licensed mammalian-specific flavivirus vaccine strain with an attenuation phenotype, such as YFV 17D, JEV LAV SA14–14–2, or DENV serotypes [53]. Insect-specific flaviviruses are also used as the backbone. Subsequently, the chimeric vaccines retain the highly attenuated phenotype of backbone viruses. The chimera vaccines also maintain a low-risk risk of reversion to virulent wild-type strains compared to the engineering attenuated mutations in LAVs [54,55]. Despite potential concerns of cross-reactive immunity against backbone virus, the chimera vaccines often provide dual protection against both target and backbone viruses challenge due to the induction of T and B cell responses against both [56,57].

#### 5.1.1. Chimeric LAVs with Mammalian-Specific Flavivirus Backbone

Functional analysis suggests that prM-E genes are the main determinants of maintaining the high stability of ZIKV. Several chimeric ZIKV vaccines have been developed to include ZIKV prM/E in the genetic background of YFV, JEV, or DENV [58,59]. For example, the chimeric DENV-2 with ZIKV prM-E genes was highly attenuated in A129 mice, induced robust neutralizing antibody response, and fully protected mice from wild-type ZIKV infection [60]. The vaccine has been further evaluated in phase 1 clinical trials in flavivirus-naïve adult individuals [61]. Another example: the ChimeriVax-Zika (CYZ) was generated by employing the YFV-17D backbone and replacing its prM-E genes with those of ZIKV. CYZ was highly attenuated in mice but elicited high titers of neutralizing antibodies following one single dose vaccination in immunocompetent and A129 mice and protected mice from wild-type ZIKV challenge [62]. Interestingly, YF-ZIKprM/E offers dual protection against ZIKV as well as lethal YFV challenge following a single dose via induction of YFV-specific CD8^+^ T cell responses [56]. The ChinZIKV is a chimeric ZIKV vaccine that uses a JEV LAV (SA14–14–2) as the backbone but replaces it with prM-E genes from those of ZIKV. The vaccine was tested in mice and rhesus macaque models and was found to protect the host from subsequent lethal wild-type ZIKV challenges and prevent maternal transmission to the fetus [63]. Lastly, VacDZ was developed using the clinically validated DENV serotype-2 (DENV-2) derivative, PDK-53, as the backbone. The candidate chimeric vaccine induces neutralizing antibodies and a strong T helper 1 response in AG129 mice and protects mice from lethal wild-type ZIKV infection [54].

#### 5.1.2. Chimeric LAVs with Insect-Specific Flavivirus Backbone

A different ZIKV chimeric vaccine using an insect-specific flavivirus, the Binjari virus (BinJV), was developed by replacing the BinJ prM-E with those from ZIKV. A single dose of this attenuated virus was able to trigger strong neutralizing antibody responses and protect IFN-deficient mice from the ZIKV-induced viremia and weight loss 15 months following vaccination [64]. Aripo virus (ARPV), a novel insect-specific flavivirus, induces robust type 1 IFN response in mouse macrophages despite its defective replication in mammalian cells [65]. ARPV/ZIKV containing the precursor PrM/E genes of ZIKV in replacement of the ARPV homologs retains the vertebrate host-restriction of ARPV. A single dose of this candidate vaccine elicited strong neutralizing antibodies, ZIKV-specific CD4^+^ and CD8^+^ T cell responses and offered complete protection against ZIKV-induced diseases and in utero transmission in both wild-type immunocompetent and IFN deficient (AB6) mice [66].

### 5.2. LAV Mutants

A site-directed mutagenesis approach can generate attenuated mutants with deletions or substitutions at different sites of the ZIKV genome, including UTR region, structural, and NS genes.

#### 5.2.1. UTR Deletion Mutants

Using an infectious cDNA clone of pre-epidemic ZIKV Cambodian strain FSS13025, several mutant viruses containing 3′UTR 10-to-30-nucleotide deletions were produced to attenuate ZIKV replication via reducing viral RNA synthesis and increasing vulnerability to type-1 IFN inhibition. All mutant deletion viruses displayed smaller infectious foci, slower RNA replication, and lower peak titers compared to the wild-type ZIKV strain. Among the mutants, the virus with 3′UTR 10 nucleotide deletions (10-del ZIKV) produced the lowest viremia in mice but induced a neutralizing antibody response comparable to other mutants and wild-type ZIKV in A129 mice. A single dose from the 10-del ZIKV LAV provides complete protection from viremia by the induction of a high level of neutralizing antibodies and strong T cell responses and preventing a decrease in the sperm count in A129 mice [49]. In another study, a ZIKV LAV mutant with 20-nucleotide deletion in the 3′UTR (20-del ZIKV) also protected mice from viral transmission during pregnancy and testis damage and ZIKV infection in NHPs [67]. Furthermore, the engineered 3′UTR deletions remain stable in the LAVs after 10 rounds of continuous culturing on Vero cells, typically used for flavivirus vaccine production, and three rounds of infections in the IFN-deficient mice [67].

#### 5.2.2. LAVs with Mutations in the Structural Gene(s)

ZIKV E protein is responsible for virus entry and a determinant for viral pathogenesis. The ZIKV E-N154Q mutant was generated to contain a substitution at the E glycosylation. It is highly attenuated in mice. A single dose vaccination with the E mutant virus developed robust neutralizing antibodies and completely protected mice from wild-type ZIKV challenge. The mutant virus also exhibited diminished oral infectivity for Aedes aegypti, which is the major vector for ZIKV transmission, though it retains neurovirulence in mice [68].

#### 5.2.3. LAVs with Mutations in the NS Gene(s)

The NS4B protein has extensive homology between flaviviruses and is known to be involved in virus replication and evasion of innate host immunity [69,70,71,72,73]. The amino acid 35 to amino acid 60 regions of the N-terminal domain of NS4B protein are associated with NS4B antagonist activities for antiviral innate cytokine signaling [70,71,72]. The central portion of the NS4B protein ranging from amino acid 95 to amino acid 120 is related to flavivirus virulence phenotype as mutations in this region lead to the attenuated phenotype in YFV, JEV, and DENV. WNV NS4B-C102S, in the central hydrophobic region, was highly attenuated for both neuroinvasiveness and neurovirulence in mice [74]. In one prior study [48], by using site-directed mutagenesis, mutant strains with genetic substitutions at NS4B P36 or NS4B C100 sites were generated. Among these mutants, the ZIKV NS4B-C100S mutant was found to be most attenuated in mice and induced more potent anti-viral innate and adaptive immune responses than the parent WT ZIKV FSS13025 strain and the 10-del ZIKV LAV and thus provided protection against lethal wild-type ZIKV challenge.

#### 5.2.4. LAVs with Combinatorial Mutations in Both Structural and NS Genes

To increase the potential of genetic stability and ultimately improve the safety of ZIKV LAV single mutants, in a recent study, we developed a LAV vaccine candidate (ZE4B-36) with combined mutations in both E glycosylation and NS4B P36. The vaccine induces robust ZIKV-specific memory B cell, neutralizing antibody, and T cell-mediated immune responses and protects IFN-deficient mice from both ZIKV-induced diseases and vertical transmission. Notably, the dual mutations offer strong genetic stability as the attenuating mutations in the E and NS4B proteins are retained during serial cell culture passages. Unlike the single LAV mutant, ZE4B-36 displays a significantly reduced neuroinvasiveness and neurovirulence in addition to its low infectivity in mosquitoes [47]. Thus, combination mutations in structural and NS proteins contribute to significantly increased safety in ZIKV LAV. Ye et al. reported another ZIKV LAV, rGZ02a, which was based on three amino acid alterations in the E, NS1, and NS5 proteins. The rGZ02a mutant induces robust antibody responses with long-term durability and protects neonates from ZIKV-caused neurological disorders and brain damage [75].

### 5.3. Codon Pair Deoptimization

ZIKV can also be attenuated by codon pair deoptimization. Three codon pair-deoptimized ZIKVs (Min E, Min NS1, and Min E+NS1) were de novo synthesized and recovered by reverse genetics to contain large amounts of underrepresented codon pairs in the E and/or NS1 genes. All three variants were found to have reduced virulence in mice. The Min E+NS1 strain induced more robust neutralizing antibody responses following one single vaccination and subsequently protected mice against lethal wild-type ZIKV challenge and vertical ZIKV transmission. The codon pair deoptimization approach also reduces the risk of reversion to wild-type virulence [76].

## 6. Other Platforms of ZIKV Vaccines

In addition to LAVs, several other platforms have been pursued for ZIKV vaccine development, including the purified inactivated virus, DNA plasmid, mRNA, adenovirus vector, and measles virus vector. Here, we summarized recent findings and pinpointed the strengths and potential weaknesses of these vaccine candidates (Table 1).

Inactivated vaccines: Inactivated viral vaccines induce immune responses to multiple viral antigens. One drawback of these vaccines is that they usually require an adjuvant and multiple dosages in order to increase the magnitude of the immune response. In addition, the inactivation procedure may affect certain epitopes, which leads to a sub-neutralizing antibody response [77]. The Walter Reed Army Institute (WRAIR) developed a purified formalin-inactivated ZIKV vaccine candidate, ZPIV, which derives from a 2015 Puerto Rican ZIKV strain (PRVABC59). ZPIV induces strong antibody responses and protects from viremia following the challenge with wild-type ZIKV-strains in immunocompetent mice and NHPs [78,79]. Sanofi Pasteur (SP) further optimized ZPIV on the production and purification condition; ZPIV-SP subsequently elicited sustained neutralizing antibodies, and ZIKV-specific T and memory B-cells, thus providing complete protection against wild-type ZIKV challenge in cynomolgus macaques [79]. In the phase I clinical trial, two dosages of the inactivated vaccine (also called TAK-426) adjuvanted with aluminum hydroxide was reported to be well tolerated, and immunogenic in both flavivirus-naive and flavivirus-primed healthy adults participants [80].

**Table 1:** Summary of different platforms of ZIKV vaccine candidates.

Vaccine Platform/Status	Strengths	Weaknesses	References
LAV: Preclinical studies	Induce strong immune response Long term immunity Adjuvant not required Preservation of native antigen Rapid and durable immunity Single dose	Consideration for genetic stability	[47,48,49,58,59,60,61,62,63,64,65,66,67,68,74,75,76]
Purified inactivated vaccine: Preclinical studies, phase 1 clinical trials	Induce strong immune response to multiple viral antigens Easy to prepare	Potential epitope alteration by inactivation process Adjuvant and multiple doses are required	[78,79,80]
Recombinant DNA vaccine: Preclinical studies, clinical trials	Non-infectious Easier to design, inexpensive Low risk Stable at ambient temperature	Induce lower immunogenicity May need special device/technology for administration Potential risk of integration into the host genome	[44,81,82,83,84,85]
mRNA vaccines: Preclinical studies, phase 1 clinical trials	Highly safe Easy and fast to produce Induce strong neutralizing antibodies	Induce lower immunogenicity Potential risk of RNA-induced interferon response Need low temperature for storage Multiple doses are required	[45,46]
Viral vector vaccines: Preclinical studies, clinical trials	Induce strong immune response No adjuvant required	Risk of genomic integration Multiple doses are required Host immunity against the vector may negatively affect the effectiveness of the vaccine	[18,78,86,87,88,89,90]

DNA plasmid vaccine: A DNA vaccine is a plasmid construct containing coding DNA sequences for virus-specific antigens, a transcription promoter, and a polyadenylation sequence that facilitates protein translation. DNA vaccines do not contain infectious materials like LAVs and thus have a low risk for immunocompromised individuals. They are also stable at ambient temperature and inexpensive to produce, but they require multiple dosages. There are three DNA ZIKV vaccine candidates in the clinical trials. These include the VRC5283 and VRC5288 vaccines developed by the National Institute of Allergy and Infectious Diseases and the GLS-5700 vaccine developed by GeneOne Life Science, and they are all constructed based on prM-E antigens. Although they are genetically similar, the E protein produced by VRC5288 is chimeric, with the extracellular region being of ZIKV origin and the stem and transmembrane regions from JEV. Phase I clinical trials suggest these vaccines are well tolerated in healthy adults. The VRC5283 induces more potent neutralization antibody and T cell responses than the VRC5288 vaccine four weeks post-vaccination [44,81]. The GLS-5700 DNA ZIKV vaccine was reported to elicit neutralizing antibodies in only 60% of the participants [82]. In addition to the structural proteins, DNA-based vaccines expressing NS1 were reported to provide strong protection against ZIKV-induced viral infection and virus-induced weight loss via induction of NS1-specific T-cell responses. Compared to the PrM-E-based DNA vaccines, the NS1-based DNA vaccines do not induce neutralization antibodies [83,84]. However, these vaccines may be less risky in triggering antibody-dependent enhancement (ADE) for vaccinees living in areas endemic for DENV and other flaviviruses, as anti-NS1 antibodies do not enhance viral uptake in vitro [85].

mRNA Vaccines: They can be a promising alternative to conventional vaccine platforms due to their high potency and capacity for rapid development. Two doses of modified mRNA lipid nanoparticles (LNP) encoding the full-length ZIKV prM-E genes induced high neutralizing antibody titers in immunocompetent mice and immunocompromised mice and protected the host from wild-type ZIKV infection. To reduce cross-reactive antibody responses to enhance ADE, the modified prM-E RNA vaccine was engineered to include mutations destroying the conserved fusion-loop epitope in the E protein [46]. Currently, ZIKV mRNA-1325 (NCT03014089) and mRNA-1839 (NCT04064905) vaccine candidates developed by Moderna have progressed to phase I clinical trials. These vaccine constructs incorporate prM/E genes of ZIKV; however, they are different in signal peptides at the amino terminus of prM. mRNA-1325 expresses the signal sequence from human IgE upstream of prM [46]. Compared to the chimeric LAVs, the mRNA vaccines do not induce the anti-vector immunity associated with the chimeric vaccines. However, multiple doses of mRNA vaccine are generally needed to induce a durable immune response for host protection against the WT ZIKV challenge [45].

Viral vector vaccine: Recombinant adenovirus vectors (Ad) vectors demonstrate high transduction efficiency and induce strong innate immune responses, rendering the vector a viable option for vaccine studies [86]. Viral vector vaccines can enhance immunogenicity and induce strong cytotoxic T lymphocyte responses in the absence of an adjuvant, which results in viral clearance (28). There are several studies using different Adenovirus serotypes, including but not limited to Ad4, Ad5, Ad26, rhesus monkey Ad52, chimpanzee Ad7, and gorilla Ad. One study reported that the Ad4 vector expressing ZIKV prM-E genes led to strong T-cell responses without anti-ZIKV antibodies, while the Ad5-prM-E vaccine was able to induce both anti-ZIKV T-cell and antibody responses. The study showed that the Ad5-prM-E vector vaccine induced stronger cellular and humoral responses when compared to the Ad4 vector [87]. Another vector vaccine, ChAdOx1, was developed using a chimpanzee adenovirus vector. The study found that the addition of prM and deletion of the transmembrane domain from the ZIKV Env were optimal in promoting protective immunity, including high neutralizing antibodies without induction of antibody-dependent enhancement to DENV [88,89]. More recently, live measles vaccine (MV) vectors expressing ZIKV-E and -NS1 provided complete clearance of ZIKV from the female reproductive tract and full fetal protection in the lethal African challenge model in animals [18]. Lastly, vesicular stomatitis virus (VSV) and mutated VSV (VSVm) containing either ZIKV E protein (ZENV) alone or ZIKV prM-E proteins were also developed. The VSVm–ZprME candidate induces a strong antibody response and generated high levels of T helper 1 responses [90]. Several vector-based candidate vaccines have also been tested in NHPs, and some of them have progressed to phase 1 clinical trials [78].

## 7. Summary and Future Perspectives

The development of a safe and efficacious ZIKV vaccine remains a global health priority. Although successful efforts have led to the development of multiple other ZIKV vaccine platforms, including purified inactivated virus, plasmid DNA, mRNA, and viral vector, which all elicit high neutralizing antibodies and protect against ZIKV viremia in animal models or clinical trials, each candidate has its inherent weaknesses (Table 1). LAVs have been very successful in the control of YFV and JEV infections. Compared with other vaccine platforms, the LAV approach has the advantage of a single dose, being close to natural viral infection, the induction of rapid and durable protection, and low cost. Furthermore, the potential concerns of LAVs on genetic stability or reversion to wild-type strains can be overcome via multiple approaches, including using an insect-specific flavivirus as the backbone in chimeric LAVs, generating combinatorial mutations in both structural and NS genes in LAV mutants, or using codon pair deoptimization. Future clinical studies will further test the efficacy of these modified new-generation LAV candidates. Furthermore, for infectious diseases like ZIKV in developing countries, it is essential to have vaccines with single-dose efficacy and long-lasting immune protection. Multi-dose vaccines are practically impossible to implement in remote areas within underdeveloped countries. Combined together, LAV is a more attractive vaccine platform than others in future ZIKV vaccine development.

## Data Availability

Data sharing not applicable. No new data were created or anaylzed in this study.

## References

[1] Simmonds P., Becher P., Bukh J., Gould E.A., Meyers G., Monath T., Muerhoff S., Pletnev A., Rico-Hesse R., Smith D.B. (2017). ICTV Virus Taxonomy Profile: Flaviviridae. J. Gen. Virol..

[2] Dick G.W., Kitchen S.F., Haddow A.J. (1952). Zika virus. I. Isolations and serological specificity. Trans. R. Soc. Trop. Med. Hyg..

[3] Duffy M.R., Chen T.H., Hancock W.T., Powers A.M., Kool J.L., Lanciotti R.S., Pretrick M., Marfel M., Holzbauer S., Dubray C. (2009). Zika virus outbreak on Yap Island, Federated States of Micronesia. N. Engl. J. Med..

[4] Cao-Lormeau V.M., Roche C., Teissier A., Robin E., Berry A.L., Mallet H.P., Sall A.A., Musso D. (2014). Zika virus, French polynesia, South pacific, 2013. Emerg. Infect. Dis..

[5] Foy B.D., Kobylinski K.C., Chilson Foy J.L., Blitvich B.J., Travassos da Rosa A., Haddow A.D., Lanciotti R.S., Tesh R.B. (2011). Probable non-vector-borne transmission of Zika virus, Colorado, USA. Emerg. Infect. Dis..

[6] Sakkas H., Bozidis P., Giannakopoulos X., Sofikitis N., Papadopoulou C. (2018). An Update on Sexual Transmission of Zika Virus. Pathogens.

[7] Chen L.H., Wilson M.E. (2016). Update on non-vector transmission of dengue: Relevant studies with Zika and other flaviviruses. Trop. Dis. Travel Med. Vaccines.

[8] Adam A., Jassoy C. (2021). Epidemiology and Laboratory Diagnostics of Dengue, Yellow Fever, Zika, and Chikungunya Virus Infections in Africa. Pathogens.

[9] Alonso-Palomares L.A., Moreno-Garcia M., Lanz-Mendoza H., Salazar M.I. (2018). Molecular Basis for Arbovirus Transmission by *Aedes aegypti* Mosquitoes. Intervirology.

[10] D’Ortenzio E., Matheron S., Yazdanpanah Y., de Lamballerie X., Hubert B., Piorkowski G., Maquart M., Descamps D., Damond F., Leparc-Goffart I. (2016). Evidence of Sexual Transmission of Zika Virus. N. Engl. J. Med..

[11] Mansuy J.M., Dutertre M., Mengelle C., Fourcade C., Marchou B., Delobel P., Izopet J., Martin-Blondel G. (2016). Zika virus: High infectious viral load in semen, a new sexually transmitted pathogen?. Lancet Infect. Dis..

[12] Campos G.S., Bandeira A.C., Sardi S.I. (2015). Zika Virus Outbreak, Bahia, Brazil. Emerg. Infect. Dis..

[13] Oehler E., Watrin L., Larre P., Leparc-Goffart I., Lastere S., Valour F., Baudouin L., Mallet H., Musso D., Ghawche F. (2014). Zika virus infection complicated by Guillain-Barre syndrome—Case report, French Polynesia, December 2013. Eurosurveillance.

[14] Cao-Lormeau V.M., Blake A., Mons S., Lastere S., Roche C., Vanhomwegen J., Dub T., Baudouin L., Teissier A., Larre P. (2016). Guillain-Barre Syndrome outbreak associated with Zika virus infection in French Polynesia: A case-control study. Lancet.

[15] Cauchemez S., Besnard M., Bompard P., Dub T., Guillemette-Artur P., Eyrolle-Guignot D., Salje H., Van Kerkhove M.D., Abadie V., Garel C. (2016). Association between Zika virus and microcephaly in French Polynesia, 2013–15: A retrospective study. Lancet.

[16] Ghaffar K.A., Ng L.F.P., Renia L. (2018). Fast Tracks and Roadblocks for Zika Vaccines. Vaccines.

[17] Guo M., Hui L., Nie Y., Tefsen B., Wu Y. (2021). ZIKV viral proteins and their roles in virus-host interactions. Sci. China Life Sci..

[18] Kurup D., Wirblich C., Lambert R., Diba L.Z., Leiby B.E., Schnell M.J. (2022). Measles-based Zika vaccine induces long-term immunity and requires NS1 antibodies to protect the female reproductive tract. NPJ Vaccines.

[19] Yang M., Lai H., Sun H., Chen Q. (2017). Virus-like particles that display Zika virus envelope protein domain III induce potent neutralizing immune responses in mice. Sci. Rep..

[20] Li G., Bos S., Tsetsarkin K.A., Pletnev A.G., Despres P., Gadea G., Zhao R.Y. (2019). The Roles of prM-E Proteins in Historical and Epidemic Zika Virus-mediated Infection and Neurocytotoxicity. Viruses.

[21] Sirohi D., Kuhn R.J. (2017). Zika Virus Structure, Maturation, and Receptors. J. Infect. Dis..

[22] Valente A.P., Moraes A.H. (2019). Zika virus proteins at an atomic scale: How does structural biology help us to understand and develop vaccines and drugs against Zika virus infection?. J. Venom. Anim. Toxins. Incl. Trop. Dis..

[23] Aliota M.T., Caine E.A., Walker E.C., Larkin K.E., Camacho E., Osorio J.E. (2016). Characterization of Lethal Zika Virus Infection in AG129 Mice. PLoS Negl. Trop. Dis..

[24] Dowall S.D., Graham V.A., Rayner E., Atkinson B., Hall G., Watson R.J., Bosworth A., Bonney L.C., Kitchen S., Hewson R. (2016). A Susceptible Mouse Model for Zika Virus Infection. PLoS Negl. Trop. Dis..

[25] Rossi S.L., Tesh R.B., Azar S.R., Muruato A.E., Hanley K.A., Auguste A.J., Langsjoen R.M., Paessler S., Vasilakis N., Weaver S.C. (2016). Characterization of a Novel Murine Model to Study Zika Virus. Am. J. Trop. Med. Hyg..

[26] Cugola F.R., Fernandes I.R., Russo F.B., Freitas B.C., Dias J.L., Guimaraes K.P., Benazzato C., Almeida N., Pignatari G.C., Romero S. (2016). The Brazilian Zika virus strain causes birth defects in experimental models. Nature.

[27] Miner J.J., Cao B., Govero J., Smith A.M., Fernandez E., Cabrera O.H., Garber C., Noll M., Klein R.S., Noguchi K.K. (2016). Zika Virus Infection during Pregnancy in Mice Causes Placental Damage and Fetal Demise. Cell.

[28] Adams Waldorf K.M., Stencel-Baerenwald J.E., Kapur R.P., Studholme C., Boldenow E., Vornhagen J., Baldessari A., Dighe M.K., Thiel J., Merillat S. (2016). Fetal brain lesions after subcutaneous inoculation of Zika virus in a pregnant nonhuman primate. Nat. Med..

[29] Dudley D.M., Aliota M.T., Mohr E.L., Weiler A.M., Lehrer-Brey G., Weisgrau K.L., Mohns M.S., Breitbach M.E., Rasheed M.N., Newman C.M. (2016). A rhesus macaque model of Asian-lineage Zika virus infection. Nat. Commun..

[30] Koide F., Goebel S., Snyder B., Walters K.B., Gast A., Hagelin K., Kalkeri R., Rayner J. (2016). Development of a Zika Virus Infection Model in Cynomolgus Macaques. Front. Microbiol..

[31] Li X.F., Dong H.L., Huang X.Y., Qiu Y.F., Wang H.J., Deng Y.Q., Zhang N.N., Ye Q., Zhao H., Liu Z.Y. (2016). Characterization of a 2016 Clinical Isolate of Zika Virus in Non-human Primates. EBioMedicine.

[32] Osuna C.E., Lim S.Y., Deleage C., Griffin B.D., Stein D., Schroeder L.T., Omange R., Best K., Luo M., Hraber P.T. (2016). Zika viral dynamics and shedding in rhesus and cynomolgus macaques. Nat. Med..

[33] Adams Waldorf K.M., Nelson B.R., Stencel-Baerenwald J.E., Studholme C., Kapur R.P., Armistead B., Walker C.L., Merillat S., Vornhagen J., Tisoncik-Go J. (2018). Congenital Zika virus infection as a silent pathology with loss of neurogenic output in the fetal brain. Nat. Med..

[34] Hirsch A.J., Roberts V.H.J., Grigsby P.L., Haese N., Schabel M.C., Wang X., Lo J.O., Liu Z., Kroenke C.D., Smith J.L. (2018). Zika virus infection in pregnant rhesus macaques causes placental dysfunction and immunopathology. Nat. Commun..

[35] Aliota M.T., Dudley D.M., Newman C.M., Mohr E.L., Gellerup D.D., Breitbach M.E., Buechler C.R., Rasheed M.N., Mohns M.S., Weiler A.M. (2016). Heterologous Protection against Asian Zika Virus Challenge in Rhesus Macaques. PLoS Negl. Trop. Dis..

[36] Hirsch A.J., Smith J.L., Haese N.N., Broeckel R.M., Parkins C.J., Kreklywich C., DeFilippis V.R., Denton M., Smith P.P., Messer W.B. (2017). Zika Virus infection of rhesus macaques leads to viral persistence in multiple tissues. PLoS Pathog..

[37] Bassi M.R., Kongsgaard M., Steffensen M.A., Fenger C., Rasmussen M., Skjodt K., Finsen B., Stryhn A., Buus S., Christensen J.P. (2015). CD8^+^ T cells complement antibodies in protecting against yellow fever virus. J. Immunol..

[38] Larena M., Regner M., Lee E., Lobigs M. (2011). Pivotal role of antibody and subsidiary contribution of CD8^+^ T cells to recovery from infection in a murine model of Japanese encephalitis. J. Virol..

[39] Mathews J.H., Roehrig J.T., Brubaker J.R., Hunt A.R., Allan J.E. (1992). A synthetic peptide to the E glycoprotein of Murray Valley encephalitis virus defines multiple virus-reactive T- and B-cell epitopes. J. Virol..

[40] Akondy R.S., Johnson P.L., Nakaya H.I., Edupuganti S., Mulligan M.J., Lawson B., Miller J.D., Pulendran B., Antia R., Ahmed R. (2015). Initial viral load determines the magnitude of the human CD8 T cell response to yellow fever vaccination. Proc. Natl. Acad. Sci. USA.

[41] Halstead S.B. (2017). Achieving safe, effective, and durable Zika virus vaccines: Lessons from dengue. Lancet Infect. Dis..

[42] Aid M., Abbink P., Larocca R.A., Boyd M., Nityanandam R., Nanayakkara O., Martinot A.J., Moseley E.T., Blass E., Borducchi E.N. (2017). Zika Virus Persistence in the Central Nervous System and Lymph Nodes of Rhesus Monkeys. Cell.

[43] Elong Ngono A., Vizcarra E.A., Tang W.W., Sheets N., Joo Y., Kim K., Gorman M.J., Diamond M.S., Shresta S. (2017). Mapping and Role of the CD8(+) T Cell Response during Primary Zika Virus Infection in Mice. Cell Host Microbe.

[44] Dowd K.A., Ko S.Y., Morabito K.M., Yang E.S., Pelc R.S., DeMaso C.R., Castilho L.R., Abbink P., Boyd M., Nityanandam R. (2016). Rapid development of a DNA vaccine for Zika virus. Science.

[45] Pardi N., Hogan M.J., Porter F.W., Weissman D. (2018). mRNA vaccines—A new era in vaccinology. Nat. Rev. Drug Discov..

[46] Richner J.M., Himansu S., Dowd K.A., Butler S.L., Salazar V., Fox J.M., Julander J.G., Tang W.W., Shresta S., Pierson T.C. (2017). Modified mRNA Vaccines Protect against Zika Virus Infection. Cell.

[47] Adam A., Fontes-Garfias C.R., Sarathy V.V., Liu Y., Luo H., Davis E., Li W., Muruato A.E., Wang B., Ahatov R. (2021). A genetically stable Zika virus vaccine candidate protects mice against virus infection and vertical transmission. NPJ Vaccines.

[48] Li G., Adam A., Luo H., Shan C., Cao Z., Fontes-Garfias C.R., Sarathy V.V., Teleki C., Winkelmann E.R., Liang Y. (2019). An attenuated Zika virus NS4B protein mutant is a potent inducer of antiviral immune responses. NPJ Vaccines.

[49] Shan C., Muruato A.E., Nunes B.T.D., Luo H., Xie X., Medeiros D.B.A., Wakamiya M., Tesh R.B., Barrett A.D., Wang T. (2017). A live-attenuated Zika virus vaccine candidate induces sterilizing immunity in mouse models. Nat. Med..

[50] Cohen J. (2017). Zika rewrites maternal immunization ethics. Science.

[51] Holland J., Spindler K., Horodyski F., Grabau E., Nichol S., VandePol S. (1982). Rapid evolution of RNA genomes. Science.

[52] Kenney J.L., Volk S.M., Pandya J., Wang E., Liang X., Weaver S.C. (2011). Stability of RNA virus attenuation approaches. Vaccine.

[53] Lai C.J., Monath T.P. (2003). Chimeric flaviviruses: Novel vaccines against dengue fever, tick-borne encephalitis, and Japanese encephalitis. Adv. Virus Res..

[54] Chin W.X., Lee R.C.H., Kaur P., Lew T.S., Yogarajah T., Kong H.Y., Teo Z.Y., Salim C.K., Zhang R.R., Li X.F. (2021). A single-dose live attenuated chimeric vaccine candidate against Zika virus. NPJ Vaccines.

[55] Huang C.Y., Silengo S.J., Whiteman M.C., Kinney R.M. (2005). Chimeric dengue 2 PDK-53/West Nile NY99 viruses retain the phenotypic attenuation markers of the candidate PDK-53 vaccine virus and protect mice against lethal challenge with West Nile virus. J. Virol..

[56] Kum D.B., Boudewijns R., Ma J., Mishra N., Schols D., Neyts J., Dallmeier K. (2020). A chimeric yellow fever-Zika virus vaccine candidate fully protects against yellow fever virus infection in mice. Emerg. Microbes Infect..

[57] Mishra N., Boudewijns R., Schmid M.A., Marques R.E., Sharma S., Neyts J., Dallmeier K. (2020). A Chimeric Japanese Encephalitis Vaccine Protects against Lethal Yellow Fever Virus Infection without Inducing Neutralizing Antibodies. mBio.

[58] Annamalai A.S., Pattnaik A., Sahoo B.R., Guinn Z.P., Bullard B.L., Weaver E.A., Steffen D., Natarajan S.K., Petro T.M., Pattnaik A.K. (2019). An Attenuated Zika Virus Encoding Non-Glycosylated Envelope (E) and Non-Structural Protein 1 (NS1) Confers Complete Protection against Lethal Challenge in a Mouse Model. Vaccines.

[59] Annamalai A.S., Pattnaik A., Sahoo B.R., Muthukrishnan E., Natarajan S.K., Steffen D., Vu H.L.X., Delhon G., Osorio F.A., Petro T.M. (2017). Zika Virus Encoding Nonglycosylated Envelope Protein Is Attenuated and Defective in Neuroinvasion. J. Virol..

[60] Xie X., Yang Y., Muruato A.E., Zou J., Shan C., Nunes B.T., Medeiros D.B., Vasconcelos P.F., Weaver S.C., Rossi S.L. (2017). Understanding Zika Virus Stability and Developing a Chimeric Vaccine through Functional Analysis. mBio.

[61] https://www.niaid.nih.gov/diseases-conditions/zika-vaccines.

[62] Giel-Moloney M., Goncalvez A.P., Catalan J., Lecouturier V., Girerd-Chambaz Y., Diaz F., Maldonado-Arocho F., Gomila R.C., Bernard M.C., Oomen R. (2018). Chimeric yellow fever 17D-Zika virus (ChimeriVax-Zika) as a live-attenuated Zika virus vaccine. Sci. Rep..

[63] Li X.F., Dong H.L., Wang H.J., Huang X.Y., Qiu Y.F., Ji X., Ye Q., Li C., Liu Y., Deng Y.Q. (2018). Development of a chimeric Zika vaccine using a licensed live-attenuated flavivirus vaccine as backbone. Nat. Commun..

[64] Hazlewood J.E., Tang B., Yan K., Rawle D.J., Harrison J.J., Hall R.A., Hobson-Peters J., Suhrbier A. (2022). The Chimeric Binjari-Zika Vaccine Provides Long-Term Protection against ZIKA Virus Challenge. Vaccines.

[65] Auguste A.J., Langsjoen R.M., Porier D.L., Erasmus J.H., Bergren N.A., Bolling B.G., Luo H., Singh A., Guzman H., Popov V.L. (2021). Isolation of a novel insect-specific flavivirus with immunomodulatory effects in vertebrate systems. Virology.

[66] Porier D.L., Wilson S.N., Auguste D.I., Leber A., Coutermarsh-Ott S., Allen I.C., Caswell C.C., Budnick J.A., Bassaganya-Riera J., Hontecillas R. (2021). Enemy of My Enemy: A Novel Insect-Specific Flavivirus Offers a Promising Platform for a Zika Virus Vaccine. Vaccines.

[67] Shan C., Muruato A.E., Jagger B.W., Richner J., Nunes B.T.D., Medeiros D.B.A., Xie X., Nunes J.G.C., Morabito K.M., Kong W.P. (2017). A single-dose live-attenuated vaccine prevents Zika virus pregnancy transmission and testis damage. Nat. Commun..

[68] Fontes-Garfias C.R., Shan C., Luo H., Muruato A.E., Medeiros D.B.A., Mays E., Xie X., Zou J., Roundy C.M., Wakamiya M. (2017). Functional Analysis of Glycosylation of Zika Virus Envelope Protein. Cell Rep..

[69] Munoz-Jordan J.L., Laurent-Rolle M., Ashour J., Martinez-Sobrido L., Ashok M., Lipkin W.I., Garcia-Sastre A. (2005). Inhibition of alpha/beta interferon signaling by the NS4B protein of flaviviruses. J. Virol..

[70] Liu W.J., Wang X.J., Mokhonov V.V., Shi P.Y., Randall R., Khromykh A.A. (2005). Inhibition of interferon signaling by the New York 99 strain and Kunjin subtype of West Nile virus involves blockage of STAT1 and STAT2 activation by nonstructural proteins. J. Virol..

[71] Evans J.D., Seeger C. (2007). Differential effects of mutations in NS4B on West Nile virus replication and inhibition of interferon signaling. J. Virol..

[72] Munoz-Jordan J.L., Sanchez-Burgos G.G., Laurent-Rolle M., Garcia-Sastre A. (2003). Inhibition of interferon signaling by dengue virus. Proc. Natl. Acad. Sci. USA.

[73] Lundin M., Monne M., Widell A., Von Heijne G., Persson M.A. (2003). Topology of the membrane-associated hepatitis C virus protein NS4B. J. Virol..

[74] Wicker J.A., Whiteman M.C., Beasley D.W., Davis C.T., Zhang S., Schneider B.S., Higgs S., Kinney R.M., Barrett A.D. (2006). A single amino acid substitution in the central portion of the West Nile virus NS4B protein confers a highly attenuated phenotype in mice. Virology.

[75] Ye X., Liu X., Shu T., Deng W., Liao M., Zheng Y., Zheng X., Zhang X., Li T., Fan W. (2021). A Live-Attenuated Zika Virus Vaccine with High Production Capacity Confers Effective Protection in Neonatal Mice. J. Virol..

[76] Li P., Ke X., Wang T., Tan Z., Luo D., Miao Y., Sun J., Zhang Y., Liu Y., Hu Q. (2018). Zika Virus Attenuation by Codon Pair Deoptimization Induces Sterilizing Immunity in Mouse Models. J. Virol..

[77] Murphy B.R., Walsh E.E. (1988). Formalin-inactivated respiratory syncytial virus vaccine induces antibodies to the fusion glycoprotein that are deficient in fusion-inhibiting activity. J. Clin. Microbiol..

[78] Abbink P., Stephenson K.E., Barouch D.H. (2018). Zika virus vaccines. Nat. Rev. Microbiol..

[79] Lecouturier V., Pavot V., Berry C., Donadieu A., de Montfort A., Boudet F., Rokbi B., Jackson N., Heinrichs J. (2020). An optimized purified inactivated Zika vaccine provides sustained immunogenicity and protection in cynomolgus macaques. NPJ Vaccines.

[80] Han H.H., Diaz C., Acosta C.J., Liu M., Borkowski A. (2021). Safety and immunogenicity of a purified inactivated Zika virus vaccine candidate in healthy adults: An observer-blind, randomised, phase 1 trial. Lancet Infect. Dis..

[81] Gaudinski M.R., Houser K.V., Morabito K.M., Hu Z., Yamshchikov G., Rothwell R.S., Berkowitz N., Mendoza F., Saunders J.G., Novik L. (2018). Safety, tolerability, and immunogenicity of two Zika virus DNA vaccine candidates in healthy adults: Randomised, open-label, phase 1 clinical trials. Lancet.

[82] Tebas P., Roberts C.C., Muthumani K., Reuschel E.L., Kudchodkar S.B., Zaidi F.I., White S., Khan A.S., Racine T., Choi H. (2017). Safety and Immunogenicity of an Anti-Zika Virus DNA Vaccine—Preliminary Report. N. Engl. J. Med..

[83] Grubor-Bauk B., Wijesundara D.K., Masavuli M., Abbink P., Peterson R.L., Prow N.A., Larocca R.A., Mekonnen Z.A., Shrestha A., Eyre N.S. (2019). NS1 DNA vaccination protects against Zika infection through T cell-mediated immunity in immunocompetent mice. Sci. Adv..

[84] Zhan Y., Pang Z., Du Y., Wang W., Yang Y., Wang W., Gao G.F., Huang B., Deng Y., Tan W. (2020). NS1-based DNA vaccination confers mouse protective immunity against ZIKV challenge. Infect. Genet. Evol..

[85] Bailey M.J., Duehr J., Dulin H., Broecker F., Brown J.A., Arumemi F.O., Bermudez Gonzalez M.C., Leyva-Grado V.H., Evans M.J., Simon V. (2018). Human antibodies targeting Zika virus NS1 provide protection against disease in a mouse model. Nat. Commun..

[86] Wold W.S., Toth K. (2013). Adenovirus vectors for gene therapy, vaccination and cancer gene therapy. Curr. Gene Ther..

[87] Bullard B.L., Corder B.N., Gordon D.N., Pierson T.C., Weaver E.A. (2020). Characterization of a Species E Adenovirus Vector as a Zika virus vaccine. Sci. Rep..

[88] Lopez-Camacho C., Abbink P., Larocca R.A., Dejnirattisai W., Boyd M., Badamchi-Zadeh A., Wallace Z.R., Doig J., Velazquez R.S., Neto R.D.L. (2018). Rational Zika vaccine design via the modulation of antigen membrane anchors in chimpanzee adenoviral vectors. Nat. Commun..

[89] Dejnirattisai W., Supasa P., Wongwiwat W., Rouvinski A., Barba-Spaeth G., Duangchinda T., Sakuntabhai A., Cao-Lormeau V.M., Malasit P., Rey F.A. (2016). Dengue virus sero-cross-reactivity drives antibody-dependent enhancement of infection with zika virus. Nat. Immunol..

[90] Betancourt D., de Queiroz N.M., Xia T., Ahn J., Barber G.N. (2017). Cutting Edge: Innate Immune Augmenting Vesicular Stomatitis Virus Expressing Zika Virus Proteins Confers Protective Immunity. J. Immunol..

